# Automated Analysis of Domestic Violence Police Reports to Explore Abuse Types and Victim Injuries: Text Mining Study

**DOI:** 10.2196/13067

**Published:** 2019-03-12

**Authors:** George Karystianis, Armita Adily, Peter W Schofield, David Greenberg, Louisa Jorm, Goran Nenadic, Tony Butler

**Affiliations:** 1 The Kirby Institute Faculty of Medicine The University of New South Wales Sydney Australia; 2 Neuropsychiatry Service Hunter New England Health Newcastle Australia; 3 School of Psychiatry The University of New South Wales Sydney Australia; 4 Centre for Big Data Research in Health The University of New South Wales Sydney Australia; 5 School of Computer Science The University of Manchester Manchester United Kingdom

**Keywords:** domestic violence, injuries, abuse types, text mining, rule-based approach, police narratives

## Abstract

**Background:**

The police attend numerous domestic violence events each year, recording details of these events as both structured (coded) data and unstructured free-text narratives. Abuse types (including physical, psychological, emotional, and financial) conducted by persons of interest (POIs) along with any injuries sustained by victims are typically recorded in long descriptive narratives.

**Objective:**

We aimed to determine if an automated text mining method could identify abuse types and any injuries sustained by domestic violence victims in narratives contained in a large police dataset from the New South Wales Police Force.

**Methods:**

We used a training set of 200 recorded domestic violence events to design a knowledge-driven approach based on syntactical patterns in the text and then applied this approach to a large set of police reports.

**Results:**

Testing our approach on an evaluation set of 100 domestic violence events provided precision values of 90.2% and 85.0% for abuse type and victim injuries, respectively. In a set of 492,393 domestic violence reports, we found 71.32% (351,178) of events with mentions of the abuse type(s) and more than one-third (177,117 events; 35.97%) contained victim injuries. “Emotional/verbal abuse” (33.46%; 117,488) was the most common abuse type, followed by “punching” (86,322 events; 24.58%) and “property damage” (22.27%; 78,203 events). “Bruising” was the most common form of injury sustained (51,455 events; 29.03%), with “cut/abrasion” (28.93%; 51,284 events) and “red marks/signs” (23.71%; 42,038 events) ranking second and third, respectively.

**Conclusions:**

The results suggest that text mining can automatically extract information from police-recorded domestic violence events that can support further public health research into domestic violence, such as examining the relationship of abuse types with victim injuries and of gender and abuse types with risk escalation for victims of domestic violence. Potential also exists for this extracted information to be linked to information on the mental health status.

## Introduction

### Background

Domestic violence is a global social and public health phenomenon with important health consequences that affect thousands of lives each year [1-3]. It can be defined as “any incident of threatening behavior, violence (or psychological, physical, sexual, financial, emotional) abuse between adults who are or have been an intimate partner or family member, regardless of gender or sexuality” [4-6]. However, domestic violence can also occur in other relationship structures such as between a caregiver and a dependent person, including a child, or those living together in a household but not in an intimate relationship [4,5]. A multicountry violence study conducted by the World Health Organization estimates a prevalence of 15%-71% in physical and sexual partner violence toward women [1,3]. In Australia, in 2018, one of six women and one of 16 men experienced physical or sexual violence by a current or previous partner [7]. Domestic violence has various forms—from physical to emotional and verbal abuse. The type of abuse received and perpetrated may vary by gender, with each type bearing short- and long-term (physical and mental) health consequences for the victims [8-11]. Domestic violence bears a significant economic cost: Within Australia alone, the cost of violence against women was around Aus $22.2 billion in 2015-2016 [2,3,12].

The New South Wales Police Force (NSWPF) recorded 123,330 domestic violence–related events in 2017 in WebCOPS (Web Computerised Operational Policing System), a Web-based interface for the COPS, which enables the police to capture and analyze crime information on an organization-wide basis [13]. WebCOPS contains detailed information about domestic violence events as both structured fields (date of birth, Aboriginal status, whether weapons were used, etc) and free unstructured text called “event narratives.” An event can contain more than one text narrative describing, in detail, alleged incident(s) that occurred between the person of interest (POI) and the victim, information regarding the circumstances of the event, and any action(s) taken by the police. Narratives are frequently written without a specific structure, featuring various misspellings, typographical and grammatical errors, and (sometimes informal) acronyms and abbreviations that can have different meanings depending on the context [13].

Domestic violence event narratives contain a wealth of important information regarding injuries and abuse types, which is not found in the medical records unless medical attention is sought, although even attainment of medical attention may not be flagged as related to domestic violence. However, the volume of the recorded data along with the associated long unstructured narratives makes it difficult to identify potentially meaningful information through traditional ethnographic/qualitative research methods involving eyeballing the records. One research paper recently commented that “...there is no systematic way to extract information from these [police] narratives other than by manual review” [14].

### Prior Work

There is a need for methods that can automatically extract information of interest from large volumes of data in a short time. Text mining has been used for more than 30 years to harvest information from unstructured text in many fields, particularly in biomedicine [15-20]. Recent efforts have sought to text mine crime-related information from online media publications [21-23], with limited attempts to process police reports [13,24-28]. Previous work extracted data on the names, narcotic drugs, and weapons with varying degrees of success (F1-score ranging from 46% to 81%) through named entity extraction [24,25] and police report classification of events as domestic violence or nondomestic violence related, using an unsupervised clustering technique that correctly classified 44% of the reports set aside for manual inspection [26]. Other efforts included recognition of crime-related information (such as drugs, weapons, and facial features) from witness narratives through dictionaries and rules, with F1-scores ranging from 82% to 93% [27,28]. Recently, Karystianis et al applied a rule-based approach combined with manually crafted dictionaries to extract mentions of mental illnesses for POIs and victims from police text narratives of recorded domestic violence events with an average F1-score of 84% [13].

### Aim

In this paper, we investigate whether the application of a text mining method can automatically extract abuse types (conducted by POIs) and sustained victim injuries from a large-scale corpus of 492,393 domestic violence events.

## Methods

### Data

We used a corpus of 492,393 domestic violence events provided to the researchers by the NSWPF, occurring from January 2005 to December 2016 [13]. The domestic violence events were flagged in WebCOPS as “domestic violence related,” the description of violence was coded as “domestic,” and the relationship between the victim and the POI included any of the following: “spouse/partner” (including ex-spouse/ex-partner), “boyfriend/girlfriend” (including ex-boyfriend/ex-girlfriend), “parent/guardian” (including step/foster), “child” (including step/foster), “sibling,” “other member of family” (including kin), or “carer.” These events covered the following categories: various types of assaults; breaches of Apprehended Violence Orders; homicides; malicious damage to property; and offense against another person such as intimidation, kidnapping, abduction, and harassment. These data included only events with recorded physical assaults and any cases with stalking, sexual assault, and young POIs were not included.

Permission to access the narratives was granted by the NSWPF following ethics approval from the University of New South Wales Human Research Ethics Committee (Ref: HC16558). Due to the inclusion of sensitive and personal information (eg, name, surname, and address) in the narratives, all processing was undertaken at the NSWPF headquarters. Only de-identified, extracted outputs were allowed to be taken offsite for further analysis.

**Figure 1:**
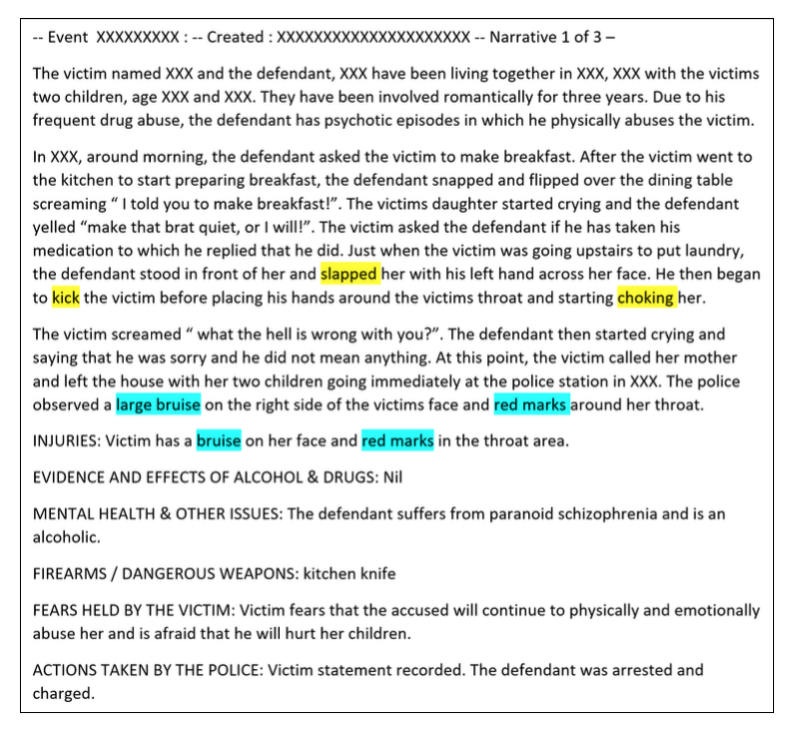
A hypothetical example of a domestic violence event narrative as recorded by the New South Wales Police Force. Blue-highlighted terms indicate the annotated victim injuries, and yellow-highlighted terms indicate the abuse types.

We used a total of 300 narratives for training, development (used to enhance the performance of the rules), and evaluation purposes (100 each). These sets are described in more detail in our previous work [13]. A hypothetical de-identified narrative is shown in Figure 1.

### Categorizing Abuse Types

We categorized specific abuse types (ie, details of the abuse behavior) using several sources into nine categories [12,29,30] with 44 abuse types (Table 1). Although the provided data did not include domestic violence events involving sexual assault and stalking, there were still cases wherein these types of abuse were described in an event. Several nonspecific forms of violence (eg, “bashing,” “smack,” “assaulted,” and “clipping”) were categorized as “assault (unspecified).” A more detailed explanation of the abuse types is provided in Multimedia Appendix 1. A total of 17 common injury types were examined, including scratching, grazing, red mark/sign, tear off (nail), bruising, cut/abrasion, swelling, lump, other, fracture, black eye, broken tooth, burn mark, stab wound, bite mark, soreness, and bleeding.

**Table 1:** Categories of abuse along with abuse types.

Abuse category	Abuse type
Physical assault	Assault (unspecified), biting, blocking, choking, ordered dog attack, dragging, elbowing, attempting to set fire to premises, gagging, grabbing, hair pulling, headbutting, head locking, kicking, kneeing, physical restraining, pulling, punching, pushing, scratching, shaking, slapping, spitting, stabbing, victim being thrown around, limb twisting, attempt to harm a victim with an object or weapon, and hitting the victim with an object or weapon
Threat	Intimidation (via body language) or stating explicit threat(s) to physically harm, sexually assault, and self-harm if the victim does not comply
Sexual assault	Sexual assault (eg, rape)
Emotional/verbal abuse	Self-harming when the victim does not comply, yelling profanities, and other emotional/verbal abuse
Stalking	Stalking, harassment, and forced entry
Financial abuse	Financial control (eg, no access to credit card)
Social abuse	Social restriction and prevent/limit child access
Unclassified	Apprehended Domestic Violence Order breach, chasing, lunging, other, and possession of personal effects (eg, phone and car keys)
Property damage	Property damage (ranging from breaking an item to causing damage to a house or vehicle)

### Rule-Based System Development

#### Overview

**Figure 2:**
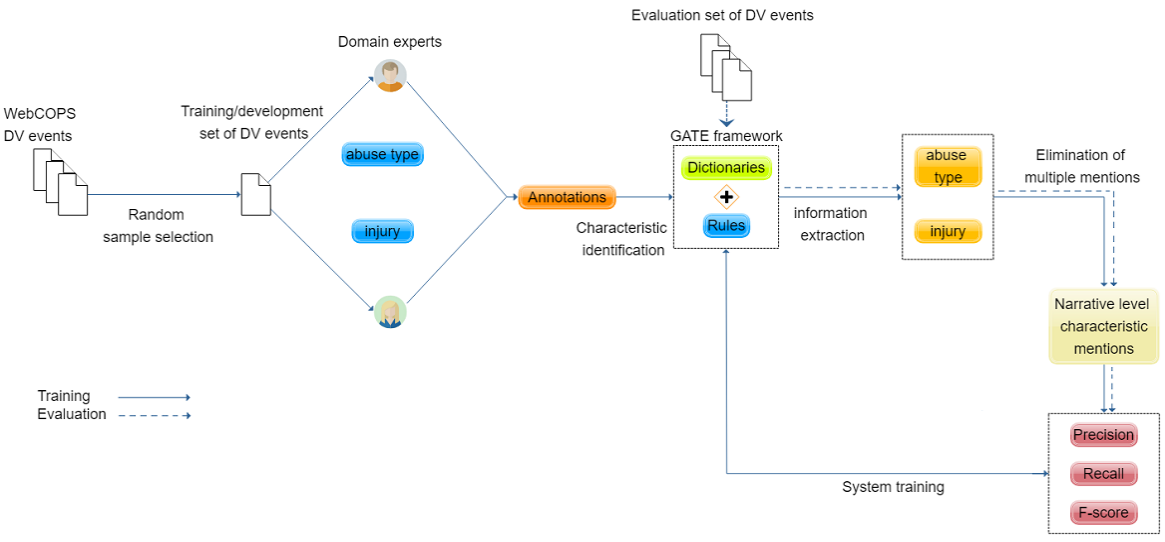
An overview of the text-mining methodology used for the identification of abuse types and victim injuries from domestic violence police event narratives. DV: domestic violence; GATE: General Architecture for Engineering; WebCOPS: Web Computerised Operational Policing System.

Our method involved the design and implementation of rule-based language expression patterns combined with dictionary terms for the recognition of abuse types and victim injuries at the narrative level. It consisted of the following steps (Figure 2): (1) creation of relevant dictionaries to recognize mentions of abuse types and victim injuries, (2) design and implementation of rules to capture abuse types and victim injuries mentions in context, and (3) aggregation of multiple mentions in each narrative to reach domestic violence event–level annotation.

#### Dictionaries

We recognized mentions of task-specific semantic groups through the development of 22 custom-made dictionaries (Table 2). The dictionaries were manually crafted by inspecting the training and the development sets for terms and expressions that describe abuse types (conducted by POIs) and victim injuries, by the first author (GK) and checked by two other authors (AA and PS) to ensure consistency. We used systematic variation (such as plural, past, and present tenses) and also included common misspellings (eg, “stuck” instead of “struck,” “harassment,” and “assalting”) frequently present in the narratives. Although the majority of the terms are noun phrases, for the “threat” dictionary, we included verbal threats made by POIs and manually expanded variations by changing a noun (eg, “your kids are going to have no *father”* to “your kids are going to have no *mother”*) and the surface expressions (*“your* dead” to *“you’re* dead” or *“you are* dead”).

**Table 2:** The manually crafted dictionaries and their respective size (number of terms included) used to identify abuse types and victim injuries.

Dictionary name	Size	Description	Examples
Anatomy	108	Anatomical parts of the human body in which a victim has been injured by the POI^a^	Chest, leg, head, neck
Assault	18	Verbs that indicate a nonspecific physical attack	Attacked, clipped, smacking, bashing
Attempt	6	Verbs that suggested a physical effort by the POI to harm the victim	Attempted, aimed, trying, tried
Be	4	Conjugations of the verb “be” in the present and past tense	Is, was, were, are
Confiscate	8	Verbs describing a confiscating act by an offender towards a victim	Confiscated, grabbed, snatched, grabbing
Damage	22	Verbs indicating an act of property damage by the POI	Cracked, burned, shuttering, ripping
Degree	14	Adjectives describing the victim’s wound	Superficial, extensive, minor, major
Description	59	Terms (mostly adjectives) describing various attributes of an object such as color or type of made material	Yellow, wooden, serving, frying
Family	31	Various nouns indicating the relationship between individuals	Boyfriend, mother, father, cousin
First person threats	123	Threats made by the POI towards a victim	“I will kill you,” “I am going to bury you,” “I will hunt you down and kill you,” “someone is going to kill you”
Force	8	Verbs describing an offender physically restrain a victim	Forcing, pinned, pinning, kept
Location	15	House locations that a DV^b^ event occurred at	Toilet, loungeroom, wall, hallway
Number	10	Numbers in words suggesting the number of criminal counts charged at an offender	One, two, four, six
Object	174	Various objects that were broken or used in a DV event	Table leg, cup, rear door, window
POI	18	Terms that describe an offender in a DV event	Defendant, person of interest (offender), offender accused
Premises	6	Terms describing a residence	Unit, terrace, flat, premises
Preposition	44	Various prepositions suggesting the presence of a victim’s injury in an anatomical part	Under left, lower, upper, front
Start	7	Verbs suggesting the initiation or continuation of an action by the offender	Begun, commenced, continuing, started
Trauma	14	Terms indicating a wound caused by a weapon/object used by the offender towards a victim	Wound, cut, trauma, fracture
Victim	19	Terms describing a victim in a DV event	Victim, vic, pinop (short for person in need for protection), pn (short for pinop),
Weapon	155	Objects used to cause harm or threaten to cause harm to a victim by an offender	Army knife, torch, book, shotgun

^a^POI: person of interest.

^b^DV: domestic violence.

#### Rules

We based our rules on syntactical patterns identified in the training and development sets, indicating the presence of an abuse type or victim injury. This work follows the same methodology that we previously developed [13]. The syntactical patterns included frozen syntactical expressions as anchors for certain elements built through specific verbs, noun phrases, and prepositions (eg, “commenced to choke”) and semantic placeholders identifiable through the application of the manually crafted dictionaries (all possible synonyms describing a victim, such as “victim,” “vic,” and “pinop”). We specifically utilized concept enumeration, since it frequently appeared in the training and development sets (eg, “Injuries: *Swollen hand*, *soreness* and *scratch under left eye* [mentions of victim’s injuries]”).

General Architecture for Text Engineering (GATE) [31], a text mining framework for annotating and categorizing text, enabling information recognition, was used to create and apply our rules. The observed syntactical patterns were converted into rules via Java Annotations Pattern Engine, GATE’s pattern-matching language. A total of 64 rules were created (Multimedia Appendix 2).

### Elimination of Multiple Mentions

More than one syntactical pattern may be matched in an event narrative and may refer to one or more mentions of abuse types of victim injuries (that can be duplicates). This led to the extraction of highly variable mentions of abuse types and victim injuries (eg, “punch,” “punched,” and “punching” are variations of the same abuse type [“punching”]; “bruised,” “bruises,” and “purple marks” are variations of the same injury [“bruising”]). Each mention is therefore mapped to its “canonical” representative, and only one mention for each abuse type or injury is kept and used to “tag” the domestic violence narrative. For example, if, in a domestic violence event report, we have extracted three mentions of the abuse type “punching” and two mentions of the abuse type “kicking,” we only annotate two abuse types—“punching” and “kicking”—at the domestic violence event level.

## Results

### Evaluation

The text mining system was evaluated on a set of 100 previously unseen, randomly chosen domestic violence event reports. The set was manually inspected and annotated by the first and second authors (GK and AA) who identified the type(s) of abuse and victim injuries. The inter-annotator agreement calculated as the absolute agreement rate [32] was 91%, suggesting reliable annotations. Performance of the methodology was evaluated at the narrative level (after eliminating any multiple characteristic mentions). We calculated the precision (the number of true positives against the number of true positives and false positives), recall (the number of true positives against the number of true positives and false negatives), and F1-score (the harmonic mean between precision and recall) at the domestic violence event level using standard definitions [33]. We defined true positive as the detection of a correct mention in an event; false positive as the extraction of any unrelated mention that has not been annotated manually; false negative as the correct mention that was not detected by our method; and true negative as the case where our method did not identify any mentions when none were annotated.

The results are shown in Table 3. Injuries and abuse types returned F1-scores above 85%, suggesting reliable and consistent results with small but expected drops from the training (5.5% and 9.6%, respectively) and development sets (3.9% and 6.7%, respectively). In particular, the precision was 90.2% for abuse types and 85.0% for the victim injuries, with a small decrease from the development set (2.6% and 5.2%, respectively). In a similar fashion, recall was 89.6% and 86.3% for the abuse types and victim injuries, respectively, with a drop of 5.2% and 8.0%, respectively, when compared to the values of the development set. However, the evaluation set had a significantly smaller number of victim injury mentions (n=66) from the development set (n=88) and the training set (n=83); therefore, its recall value should be considered with caution.

### Large-Scale Corpus Analysis

Given the relatively accurate results of the method in identifying abuse types and victims’ injuries, we applied the method to the corpus of 492,393 domestic violence events. Over 71.32% of events (351,178) had an identified abuse type as mentioned in the report, whereas more than one-third of those events (177,607; 36.07%) contained a victim injury (Table 4).

Of the 44 abuse types, “emotional/verbal abuse” (117,488; 33.46%) was the most common, followed by “punching” (86,322; 24.58%) and “property damage” (78,203; 22.27%). A total of 35.45% (124,498 events) of domestic violence events contained only one identified abuse type, whereas 33.83% (118,819 events) of domestic violence events included three to five different abuse types (Table 5).

The most frequent injury type was “bruising” (51,455; 29.03%), followed by “cut/abrasion” (51,284; 28.93%) and “red marks/signs” (42,038; 23.71%) (Table 6). A total of 105,493 domestic violence events (59.56%) had only one form of injury, and 24.48% (43,373) of domestic violence events had two forms of injury (Table 7).

**Table 3:** Performance of the system on the training, development, and evaluation sets for the identification of abuse types and victim injuries with true positive, false positive, and false negative results.

Set and characteristic		Precision (%)	Recall (%)	F1-score (%)	True positive (%)	False positive (%)	False negative (%)
**Evaluation**							
	Abuse type	90.2	89.6	89.8	259	28	30
	Injury	85.0	86.3	85.6	57	10	9
**Development**							
	Abuse type	92.8	94.8	93.7	310	24	17
	Injury	90.2	94.3	92.3	83	9	5
**Training**							
	Abuse type	93.9	96.3	95.3	293	19	11
	Injury	93.1	97.5	95.2	81	6	2

**Table 4:** Number of domestic violence events containing various abuse types (n=351,178).

Abuse type	Events, n (%)
Assault (unspecified)	171,323 (48.79)
Emotional/verbal abuse	117,488 (33.46)
Punching	86,322 (24.58)
Property damage	78,203 (22.27)
Intimidation	75,662 (21.55)
Grabbing	66,728 (19.00)
Pushing	62,794 (17.88)
Scratching	20,493 (5.84)
Physical restraining	20,014 (5.70)
Kicking	19,435 (5.53)
Slapping	17,474 (4.98)
ADVO^a^ breach	16,903 (4.81)
Attempting to hit with an object or weapon	13,592 (3.87)
Hair pulling/dragging by hair	13,048 (3.72)
Choking	11,325 (3.22)
Spitting	9341 (2.66)
Hitting with an object or weapon	8387 (2.39)
Other	7135 (2.03)
Pulling	6373 (1.81)
Victim being thrown around	5255 (1.50)
Lunging	4685 (1.33)
Possession of personal effects	3265 (0.93)
Blocking	3163 (0.90)
Harassment	3100 (0.88)
Stalking	2940 (0.84)
Self-harming	2597 (0.74)
Biting	2285 (0.65)
Dragging	2216 (0.63)
Shaking	2098 (0.60)
Stabbing	1903 (0.54)
Forced entry	1779 (0.51)
Headlocking	1482 (0.42)
Chasing	1324 (0.38)
Kneeing	1321 (0.38)
Gagging	1161 (0.33)
Elbowing	225 (0.06)
Limb twisting	173 (0.05)
Headbutting	148 (0.04)
Sexual assault	125 (0.04)
Prevent child access	91 (0.03)
Social restriction	40 (0.01)
Financial control	29 (0.01)
Attempting to set fire to premises	28 (0.01)
Ordered dog attack	1 (0.00)

^a^ADVO: Apprehended Domestic Violence Order.

**Table 5:** Domestic violence events according to the number of abuse types (n=351,178).

Number of abuse type(s)	Events, n (%)
1	124,498 (35.45)
2	89,342 (25.44)
3-5	118,819 (33.83)
6-9	17,951 (5.11)
>10	568 (0.16)
Total	351,178 (100.0)

**Table 6:** Number of events containing various injury types (n=177,607).

Injury type	Events, n (%)
Bruising	51,455 (29.03)
Cut/abrasion	51,284 (28.93)
Red mark(s)	42,038 (23.71)
Swelling	32,581 (18.38)
Soreness	26,729 (15.08)
Other	19,778 (11.16)
Bleeding	19,154 (10.81)
Fracture(s)	17,531 (9.89)
Lump	9482 (5.35)
Grazing	7305 (4.12)
Black eye(s)	2994 (1.69)
Scratching	2399 (1.35)
Bite mark(s)	2350 (1.33)
Stab wound(s)	2346 (1.32)
Burn mark(s)	1382 (0.78)
Broken tooth	620 (0.35)
Tear off nail(s)	7 (0.00)

**Table 7:** Domestic violence events according to the number of victim injury types (n=177,607).

Number of injury types	Events, n (%)
1	105,493 (59.56)
2	43,373 (24.49)
3-4	25,678 (14.49)
5-6	2484 (1.40)
≥7	89 (0.05)
Total	177,117 (100.0)

## Discussion

### Principal Results

To the best of our knowledge, this analysis represents the first attempt to capture domestic violence–related abuse and victim injuries using a large, population-level corpus of domestic violence events recorded by the police. The identification of abuse types conducted by POIs and various injuries sustained by victims in domestic violence disputes are not recorded in the structured information of the WebCOPS database fields. We therefore focused on the narrative part, where the application of our knowledge-driven approach has identified rich information and has the potential to be used for better understanding domestic violence and the development of related prevention interventions, surveillance, and reporting.

Our findings derived from text mining present a more detailed picture of the types of injuries and abuse occurring in domestic violence events. The most common abuse type in our dataset was nonphysical and involved “emotional/verbal abuse,” which is consistent with the recent findings showing that nonphysical abuse types are more prevalent than physical ones [34] and that victims of domestic violence abuse are more likely to sustain certain types of injuries such as cuts and fractures than others [34,35]. Domestic violence can also take myriad physical forms, ranging from victim intimidation to cases where serious and grievous bodily harm is caused by a specific type of abuse (eg, “punching,” “stabbing,” and “choking”), which have both short- and long-term physical and mental health consequences [9-11].

Through the recognition of various abuse types and related victim injuries, potential exists to develop prevention and intervention guidelines by linking this information to diagnostic data held by health services, so that surveillance and monitoring of the victims can be performed. There is also a possibility to track any potential timelines in which the victim was abused. Moreover, the text mining method can be updated on an ongoing basis to monitor trends and inform risk stratification algorithms, which can drive domestic violence–prevention strategies targeting specific groups.

With the inclusion of domestic violence in the WHO’s Sustainable Development Goals, the need for accurate reporting in this area will be necessary [36]. Text mining the police’s domestic violence event narratives is possibly a source of obtaining very nuanced information on this topic including the cause of the event, the potential role of mental illness and substance (ab)use in the event, the types of abuse perpetrated, injuries sustained, weapons used, and information on relationship status. This invaluable information can then be used to target prevention strategies for use by those providing prevention services to particular groups and to identify warning signs for health care providers. A recent report indicated that in Australia, from 2012-2013 to 2013-2014, one woman was killed each week and one man was killed each month as a result of violence from a current or previous partner [7]. Subsequent analyses of this rich information will aim to examine these issues and identify early warning signs of abuse and domestic violence events, which may improve assistance in preventing homicides in domestic violence settings.

### Error Analysis

Although the level of accuracy was acceptable for large-scale analysis to identify trends in domestic violence events, there were still some errors in both abuse types and victim injuries at the level of individual narrative reports. By inspecting the evaluation set, we observed that the system erroneously extracted few instances (five cases) of several POI injuries as victim injuries, since the rules were triggered for the POIs (eg, *“minor grazing to the right shoulder* [false positive for injury] of the POI”). In other instances (4 cases), victim injuries were incorrectly identified when they actually referred to property damage through ambiguous word and syntactical pattern combinations that indicated an injury (eg, “INJURIES/MEDICAL TREATMENT/DAMAGE TO PROPERTY: *Broken table leg* [false positive for victim injury]”). In 12 domestic violence events, when a victim fought back against a POI, any actions by the victim in self-defence were erroneously extracted as an abuse type (eg, “witness stepped in and *grabbed* [false positive for abuse type] the POI and *pinned him to the ground* [false positive for abuse type] until he calmed down” and “...has admitted she physically *pushed him* [false positive for abuse type] back after he *pushed* [true positive for abuse type] into her”). There were few occasions where an abuse type was recognized but had no domestic violence context (eg, “The Accused was closed inside the caged area, where he began *kicking* [false positive for abuse type] at the door and yelling at the police officers...”), while others had not occurred but were likely to happen in the future (eg, “The victim believes if she stayed at the residence she would definitely have been *bashed* [false positive for abuse type] by the accused and possibly *stabbed* [false positive for abuse type]”).

Although we engineered the rules based on generic syntactical patterns that stated victim injuries and abuse types, these rules ignored a limited number of injury mentions, since they were not explicitly stated to have been sustained by the victim (eg, *“redness* [false negative for injury] and *grazes* [false negative for injury] sighted on back, *dried blood* [false negative for injury] on lips”). Some examples (eight cases) were more implicit and required additional inference using some related terms (eg, “the POI *placed his hand in the middle of the victim's sternum and applied force* [false negative for injury] causing her pain and shortness of breath”). Cases like these were the majority of false negatives for abuse types, suggesting that abuse types such as “grabbing” and “punching” can have quite a few lexical variations in the narratives, which indicate richness of the contexts.

Additionally, injury or abuse type mentions (six cases) that were accompanied by the victim’s surname were excluded from our rule design, since there was no way to determine from the narrative who was the victim or POI without using the structured part of the record (eg, “xxx had a *bleeding nose* [false negative for injury]” and “xxx yelled *verbal abuse* [false negative for abuse type] at her”).

### Limitations

Our text mining system could have missed cases due to more specialized or explicit mentions of abuse types occurring in domestic violence events, since we based our extraction rules on the information contained in only 200 narratives. Despite incorporation of all types of abuse, there are still likely to be cases in which we probably did not identify explicit types. The relatively smaller number of injury mentions in the evaluation set (when compared to one of the abuse types) could explain the lower performance for the injuries. Nonetheless, we designed our rules based on common syntactical patterns that would attribute abuse types/injury mentions toward POIs and victims, respectively, in order to avoid the generation of false negatives; hence, our recall was higher than the precision in all three datasets. Nevertheless, this approach was able to identify the victim’s actions as types of POI’s abuse as well as POI’s injuries as those of the victim in some instances. This suggests that more specific engineered rules could address this issue. Similarly, although we included the basic and most common forms of injuries, there would be instances containing other causes of injuries or particular abuse types leading to specific injuries that probably have been excluded from our approach. Additionally, the implementation of spell-checking algorithms could assist in the identification of any misspelled abuse types or injuries and potentially elevate performance.

Our analysis of the results from the large corpus of domestic violence events is limited to the abuse types and victim’s injuries. We plan to use this information in combination with administrative data collections on mental illness to further examine the nexus between mental illness and domestic violence and explore the relationship of abuse types with gender and victim injuries. It is pertinent to inquire whether domestic violence victims with mental illness are more vulnerable than those without mental illness in this large-scale dataset spanning 10 years, to identify new intel. Further analysis of the results combined with demographic variables can show interesting aspects of the data in the area of the domestic violence, from prevalence and incidence rates in specific cohorts to risk factors for the occurrence (or recurrence) of domestic violence events. The combination of victim injuries from clinical data resulting from health service contacts could assist in the early identification of victim abuse and the implementation of intervention strategies. Modelling will be used to investigate whether POI characteristics can predict severity of abuse and similarly, whether certain victim phenotypes are prone to particular types of abuse.

### Conclusions

We demonstrated that a knowledge-driven approach can be used for the automated extraction of abuse types and victim injuries involved in domestic violence events. The performance was encouraging, with 90.2% and 85.0% precision for abuse types and injuries, respectively, further implicating that text mining can be used to extract meaningful information from these unstructured data on a large scale. The identified information has enabled us to confirm the magnitude of abuse that victims endure during domestic violence. The results can be used to support further public health research that aims to assess the profiling of POIs involved in domestic violence events and to alter existing intervention policies for victims of abuse.

## Appendix

Multimedia Appendix 1 Brief description of the extracted abuse types.

Multimedia Appendix 2 Rule examples for recognition of abuse types and victim injuries.

## Abbreviations

ADVO: Apprehended Domestic Violence Order
DV: domestic violence
GATE: General Architecture for Engineering
NSWPF: New South Wales Police Force
POI: person of interest
WebCOPS: Web Computerised Operational Policing System
