# Supplementary material for: Automated Analysis of Domestic Violence Police Reports to Explore Abuse Types and Victim Injuries: Text Mining Study
Source: J Med Internet Res. 2019 Mar 12;21(3):e13067. doi: 10.2196/13067 (PMC6434398; doi:10.2196/13067)
Supplement: Multimedia Appendix 1 [file jmir_v21i3e13067_app1.pdf]

Appendix

Table 1: Brief description of the extracted abuse types.

Characteristic	Labels	Description
Action	ADVO breach	An offender violating their apprehended domestic violence order (ADVO) or apprehended violence order (AVO).
	Assault (unspecified)	An unspecified form of attack from an offender towards a victim reported in the DV event as bashing, clipping, assaulting, smacking.
	Attempting to hit the victim with an object or weapon	An offender using an object to cause harm or pain to a victim (but did not occur).
	Attempting to set fire to premises	An offender attempted to initiate a fire inside the premises (resulting in property damage).
	Biting	An offender biting a victim.
	Blocking	An offender physically blocking with his presence an exit or entry forcing the victim to stay in the same area.
	Chasing	An offender chasing a victim.
	Choking	An offender attempting to strangle/or strangling a victim.
	Hair pulling/dragging by hair	An offender draging a victim (either by hair or other body part).
	Elbowing	An offender attacking a victim through their elbows.
	Emotional/verbal abuse	Yellling/shouting emotional/verbal abuse towards the victim
	Financial control	An offender controlling any financial resources that belong to the victim.
	Forced entry	An offender attempting to physically force their way into the victim's premises.
	Gagging	An offender gaging a victim to avoid any cries for help.
	Grabbing	An offender grabbing forcefully a victim from any body part or wearing accessory eg bag, scarf.
	Hair pulling	An offender pulling a victim by their hair.
	Harassment	An offender harassing a victim in any way eg texts, emails, appearing on the premises

	Headbutting	An offender headbutting a victim.
	Headlocking	An offender headlocking a victim.
	Hitting the victim with an object or weapon	An offender using an object to cause harm or pain to a victim.
	Intimidation	An offender intimidating in any way (physically or explicitly making a threat of any kind) a victim.
	Kicking	An offender kicking a victim.
	Kneeing	An offender kneeing a victim.
	Limb twisting	An offender twisting one or more body parts of a victim to cause pain or harm.
	Lunging	An offener lunging towards a victim.
	Other	Various unclassified actions by offenders.
	Ordered dog attack	An offender ordering his dog to attack a victim.
	Physical restraining	An offender physically restraining a victim.
	Prevent child access	An offender prohibiting a victim seeing their children.
	Property damage	An offender causing damage to a victim's property.
	Possession	An offender wrongfully possessing items belonging to a victim e.g, cellphone, car keys.
	Pulling	An offender physically pulling a victim from a location (but not dragging).
	Punching	An offender punching a victim.
	Pushing	An offender pushing a victim.
	Scratching	An offender scratching a victim.
	Self-harming	An offender using (or threatening to use) self-harm to blackmail a victim.
	Sexual assaulting	An offender attempted to sexually assault a victim.
	Shaking	An offender physically grabbing and shaking a victim.
	Slapping	An offender slapping a victim.
	Social restriction	An offender prohibiting a victim to socialise, see relatives or friends or leave the residential premises.
	Spitting	An offender spitting at a victim.

	Stabbing	An offender stabbing at a victim.
	Stalking	An offender stalking a victim.
	Victim being thrown around	An offender physically throwing the victim on the ground, floor, wall or furniture.
