# Supplementary material for: Automated Analysis of Domestic Violence Police Reports to Explore Abuse Types and Victim Injuries: Text Mining Study
Source: J Med Internet Res. 2019 Mar 12;21(3):e13067. doi: 10.2196/13067 (PMC6434398; doi:10.2196/13067)
Supplement: Multimedia Appendix 2 [file jmir_v21i3e13067_app2.pdf]

Table 2: Rule examples (using the GATE notation) for the recognition of abuse types and victim injuries. The identified mentions are highlighted in bold. The rules use non-strict token matching (lower-case or uppercase); e.g., {Token.string==~"(?i)continued"} matches "continued"; various dictionaries contain variants, abbreviations and synonyms of terms of interest, e.g. (victim) and (injuries) contain terms for victims and victim sustained injuries (see Table 2); the presence of '?' at the end of a rule component suggests its non-conditional nature (i.e., it can appear or not in the text).

<table>
<tr><td rowspan="2">Action</td><td>Example</td><td>continued</td><td>to</td><td>punch</td><td></td><td>the</td><td>victim</td></tr>
<tr><td>Rule</td><td>{Token.string==~"(?i)continued"}</td><td>({Token.string==~"(?i)to"})?</td><td>{Token.string==~"(?i)punch|punching"}</td><td>({Token.string==~"(?i)into"})?</td><td>({Token.string==~"(?i)the"})?</td><td>(victim)</td></tr>
<tr><td colspan="8"></td></tr>
<tr><td rowspan="2">Injury</td><td>Example</td><td>photographs</td><td>taken</td><td>of</td><td>the</td><td>victims</td><td>bruises</td></tr>
<tr><td>Rule</td><td>{Token.string==~"(?i)photos|photographs"}</td><td>({Token.string==~"(?i)taken"})?</td><td>{Token.string==~"(?i)of"}</td><td>{Token.string==~"(?i)the"}</td><td>(victim)</td><td>(injuries)</td></tr>
</table>
